# Risk of bias and confounding of observational studies of Zika virus infection: A scoping review of research protocols

**DOI:** 10.1371/journal.pone.0180220

**Published:** 2017-07-07

**Authors:** Ludovic Reveiz, Michelle M. Haby, Ruth Martínez-Vega, Carlos E. Pinzón-Flores, Vanessa Elias, Emma Smith, Mariona Pinart, Nathalie Broutet, Francisco Becerra-Posada, Sylvain Aldighieri, Maria D. Van Kerkhove

**Affiliations:** 1 Pan American Health Organization, Washington, D.C., United States of America; 2 Department of Chemical and Biological Sciences, Universidad de Sonora, Sonora, Mexico; 3 Escuela de Medicina, Universidad de Santander, Bucaramanga, Colombia; 4 Grupo de Investigación de Salud, Universidad de La Sabana, Bogotá, Colombia; 5 Subdirección de Producción de Guías de Práctica Clínica, Instituto de Evaluación Tecnológica en Salud (IETS), Bogotá, Colombia; 6 Yale School of Public Health, New Haven, Connecticut, United States of America; 7 Cochrane Skin Group, The University of Nottingham, Nottingham, United Kingdom; 8 World Health Organization, Geneva, Switzerland; 9 Center for Global Health, Institut Pasteur, Paris, France; Universidade Estadual de Maringa, BRAZIL

## Abstract

**Introduction:**

Given the severity and impact of the current Zika virus (ZIKV) outbreak in the Americas, numerous countries have rushed to develop research studies to assess ZIKV and its potential health consequences. In an effort to ensure that studies are comprehensive, both internally and externally valid, and with reliable results, the World Health Organization, the Pan American Health Organization, Institut Pasteur, the networks of Fiocruz, the Consortia for the Standardization of Influenza Seroepidemiology (CONSISE) and the International Severe Acute Respiratory and Emerging Infection Consortium (ISARIC) have generated six standardized clinical and epidemiological research protocols and questionnaires to address key public health questions on ZIKV.

**Methods:**

We conducted a systematic search of ongoing study protocols related to ZIKV research. We analyzed the content of protocols of 32 cohort studies and 13 case control studies for systematic bias that could produce erroneous results. Additionally we aimed to characterize the risks of bias and confounding in observational studies related to ZIKV and to propose ways to minimize them, including the use of six newly standardized research protocols.

**Results:**

Observational studies of ZIKV face an array of challenges, including measurement of exposure and outcomes (microcephaly and Guillain-Barré Syndrome). Potential confounders need to be measured where known and controlled for in the analysis. Selection bias due to non-random selection is a significant issue, particularly in the case-control design, and losses to follow-up is equally important for the cohort design.

**Conclusion:**

Observational research seeking to answer key questions on the ZIKV should consider these restrictions and take precautions to minimize bias in an effort to provide reliable and valid results. Utilization of the standardized research protocols developed by the WHO, PAHO, Institut Pasteur, and CONSISE will harmonize the key methodological aspects of each study design to minimize bias at different stages of the study. Biases need to be considered by researchers implementing the standardized protocols as well as by users of observational epidemiological studies of ZIKV.

## Introduction

Although the Zika virus (ZIKV) was first identified in the mid twentieth century on the African continent, only fourteen cases were documented in humans prior to the first large, documented epidemic outbreak on the Island of Yap in 2007 [1]. This was followed by the largest ZIKV outbreak ever previously reported in French Polynesia from October 2013 to April 2014 [2]. Since 2015, 76 countries and territories around the world have reported mosquito-borne ZIKV transmission, particularly the Americas where Brazil has been hit the hardest [3]. In February 2016 the World Health Organization declared the ZIKV outbreak a Public Health Emergency of International Concern [4].

Infection with Zika virus is asymptomatic in an estimated 50 to 80% of cases and when Zika virus does cause illness; symptoms are generally mild and self-limited [1, 5]. The most recent outbreak of ZIKV has been associated with an increase in cases of microcephaly and congenital neurological malformations and disabilities in babies [6–9] and Guillan-Barré Syndrome (GBS) and GBS-like syndrome in adults [10, 11]. A recent systematic review of the literature found sufficient evidence to conclude that ZIKV is a cause of congenital abnormalities and is a trigger of GBS [12]. However, the authors acknowledged caveats in methodological aspects, inconsistencies, and gaps in the body of evidence for both sets of conditions. ZIKV alone may not be sufficient to cause either congenital brain abnormalities or GBS and may depend on as yet uncharacterized cofactors being present.

Epidemiological studies can provide valuable information to understand the spectrum of disease ZIKV infection causes and inform potential strategies to minimize its impact. In particular, a well-designed observational study can play an important role in understanding the associations between exposure to ZIKV and disease outcomes or other health conditions [13, 14]. However, an association does not necessarily establish causation and dubious conclusions can be drawn as a result of bias and confounding in those studies. As has been done with other infectious diseases [15], it is important to assess the risk of bias and confounding in observational studies of ZIKV.

Given the severity of the current outbreak, numerous countries have quickly developed epidemiological studies to assess ZIKV and its potential health consequences [16]. In an effort to ensure that studies are comprehensive, both internally and externally valid, and with reliable results, the World Health Organization (WHO), Pan American Health Organization (PAHO), Institut Pasteur, the Consortium for the Standardization of Influenza Seroepidemiology (CONSISE), Fiocruz, the International Severe Acute Respiratory and Emerging Infection Consortium (ISARIC) and others joined efforts to harmonize current research through discussions on ongoing research on ZIKV and associated complications. In July 2016, the collaboration produced a final set of six standardized protocols for cohort, case-control, and cross-sectional studies [17–22], summarized in Table 1.

**Table 1:** Review of six standardized protocol designs for the study of ZIKV.

General Study Design	Title of Standardized Protocol	Goals of the Study	Main Outcomes of Interest	Main Biases	Ways to Minimize the Risks of Bias
Cross-sectional Study	Cross-sectional seroprevalence study of ZIKV infection in the general population [22]	To estimate the frequency of ZIKV infection among the general population in both ZIKV-exposed areas and non ZIKV-exposed areas; to identify modifiable risk factors for ZIKV infection	Estimate seroprevalence ZIKV in ZIKV-exposed areas and non ZIKV-exposed areas	Measurement of the exposure given challenges in ZIKV serologic assays and the lack of understanding of antibody kinetics in previously (flavivirus) infected individuals Selection of a representative sample of participants from the defined geographic area with or without current/previous ZIKV circulation.	Standardized definition of ZIKV infection. Standardized method for random selection of participants. Clear information on the performance of the tests used
Cohort Study	Prospective longitudinal cohort study of ZIKV-infected patients to measure the persistence of ZIKV in body fluids [21]	To assess the presence and duration of infectious ZIKV and related markers	Presence of ZIKV RNA in body fluid samples at different time points	Measurement of the exposure. Selection of a representative sample of participants. Loss to follow-up.	Standardized definition of ZIKV infection. Validated test to be used and importance of the storage and transport for culture Researchers should 1) make all reasonable efforts to maximize recruitment and follow-up; and 2) account for dropouts.
Cohort Study	Prospective longitudinal cohort study of women and newborns exposed to ZIKV or not during the course of pregnancy [20]	To describe the clinical presentation of ZIKV infection in pregnant women; to determine the risk of congenital malformation or other birth complications associated with ZIKV status and other potential risk factors	Risk and quantification of fetal abnormalities or unusual birth complications or outcomes	Measurement of the exposure. Measurement of the outcome (fetal abnormalities). Selection bias due to possible underrepresentation of ZIKV negative and asymptomatic ZIKV positive women in low resource settings Loss to follow-up due to non-live outcome of birth. Confounding due to known and unknown factors. Over adjustment bias and unnecessary adjustment	Standardized definition of ZIKV infection. Standardized and validated methods for measuring outcomes. Encourage the participation of all pregnant women–including ZIKV negative and asymptomatic ZIKV positive women. Record outcome of pregnancy (whether miscarriage, termination or live birth), including any birth defects if detected. Standardized measurement of potential confounders. Control for confounders in analysis.
Cohort Study	Prospective longitudinal cohort study of newborns and infants born to mothers exposed to ZIKV or not exposed during pregnancy [19]	To describe the clinical presentation of babies born to women exposed versus unexposed to ZIKV during pregnancy; to evaluate the development of babies with congenital malformations associated with ZIKV and other potential risk factors	Risk and quantification of fetal abnormalities or unusual birth complications or outcomes; developmental abnormalities in newborns without fetal abnormalities or birth complications	Measurement of the exposure. Measurement of the outcome (fetal abnormalities). Selection bias due to possible underrepresentation of ZIKV negative and asymptomatic ZIKV positive women. Selection bias due to possible underrepresentation of newborns without apparent abnormalities. Differential loss to follow-up based on exposure and/or outcomes. Confounding due to known and unknown factors. Over adjustment bias and unnecessary adjustment	Standardized definition of ZIKV infection. Standardized and validated methods for measuring outcomes Researchers should 1) make all reasonable efforts to maximize recruitment and follow-up; and 2) account for dropouts. Standardized measurement of potential confounders. Control for confounders in analysis.
Case-Control Study	Case-control study to assess potential risk factors related to microcephaly, including ZIKV infection during pregnancy [17]	To determine the risk of microcephaly associated with ZIKV exposure; to determine the strength of association between microcephaly and ZIKV exposure	Risk factors for microcephaly (based on INTERGROWTH standards)	Measurement of the exposure. Measurement of the outcome (microcephaly). Selection bias due to misclassification of the outcome. Selection bias due to selection of controls with different underlying disease risk to cases. Confounding due to known and unknown factors. Measurement of confounders retrospectively–recall bias.	Standardized definition of ZIKV infection. Standardized and validated methods for measuring outcomes. The standardized protocol includes matching of controls to cases by date of conception (± 10 days), birth in the same maternity ward / hospital and mother’s area of residence [17]. Standardized measurement of potential confounders for cases and controls. Control for confounders in analysis.
Case-Control Study	Case-control study to assess potential risk factors related to Guillain-Barré Syndrome (GBS), including ZIKV infection [18]	To determine risk factors for GBS; to determine the strength of association between GBS and ZIKV exposure	Risk factors for GBS	Measurement of the exposure. Measurement of the outcome (Guillan-Barré Syndrome). Selection bias due to misclassification of the outcome. Selection bias due to selection of controls with different underlying disease risk to cases. Confounding due to known and unknown factors. of confounders retrospectively–recall bias.	Standardized definition of ZIKV. Standardized and validated methods for measuring outcomes. Use community-based controls where possible. The standardized protocol includes matching for age and area of residence [18]. The protocol suggests the consideration of matching for sex on a case by case basis, depending on whether it is preferable to include sex as a separate risk factor [18].

Here, we aim to characterize the risks of bias and confounding in ZIKV observational studies and propose ways to minimize them, including the use of the standardized protocols. Biases need to be considered by researchers implementing the standardized protocols as well as by users of observational epidemiological studies of ZIKV infection.

## Methods

The PAHO, through the creation of the Zika Virus Research Platform (http://www.paho.org/zika-research/), maintains a database of research protocols and primary research studies, including both observational and experimental study designs. These records contain data elements that describe the study's purpose, recruitment status, design, eligibility criteria, and locations, as well as other key protocol details. Resources and links to additional information are inserted by the PAHO to enhance the overall usefulness of the database. Researchers, policymakers, and others can now examine features and ongoing trends of Zika virus research.

Our analysis was based off of information from case-control and cohort studies. Many of these protocols were shared with us following correspondence directly with the principle investigator. The principle investigators frequently requested that the information in their protocol remained confidential; for that reason we analyzed the protocols generally and highlighted common biases found within the selected protocols [23–26]. Many of these protocols were not the final version and changes were made beyond the protocol submitted to the PAHO Zika Virus Research Platform.

Research protocols were collected by directly liaising with a number of institutions and organizations, such as the US Centers for Disease Control and Prevention; the US National Institutes of Health; the Microcephaly Epidemic Research Group (MERG) from Pernambuco Brazil; the International Research Consortium of Dengue Risk Assessment, Management, and Surveillance (IDAMS) research group; REACTing/INSERM; the Brazil Ministry of Health; Fiocruz and maternity hospitals in Brazil.

We additionally carried out a systematic search of registered research protocols through several databases including the International Clinical Trials Registry Platform (ICTRP) in the United States and the Sistema Nacional de Ética em Pesquisa (SISNEP) in Brazil, among others (S1 File). As a final step, authors of published studies identified (using the term “Zika”) in PubMed and Embase databases were contacted directly and invited to submit information if they were planning or conducting research related to Zika virus infection. The Zika Virus Research Platform includes protocols for 32 cohort studies (16 full text protocols) and 13 case control studies that were used as a foundation to analyze potential biases in observational studies of ZIKV. We conducted a comparative analysis of the available relevant protocols and categorized the potential biases in each of the studies.

Additionally, relevant stakeholders attempted to minimize the potential biases found in observational studies through the development of the six standardized research protocols that were created during the ZIKV research consultation in May 2016 and face-to-face meeting in Mexico City, Mexico in June 2016 [16]. These meetings permitted discussions on how to best manage common biases in observational epidemiological studies of ZIKV and allowed authors to consider the potential bias found in observational research.

## Findings and discussion

Our searches in the PAHO’s Zika Virus Research Platform found 317 research protocol titles. We applied the inclusion criteria to the titles and summaries (when available) of all records and excluded 288 of them. We contacted researchers to obtain the full text of each one of the 32 cohort and 13 case control studies that were identified. Supporting information 1–2 show our screening and study selection process of the 16 cohort protocols and 13 case control studies that were finally reviewed can be found in S2 Fig and S1 File. PRISMA 2009 Checklist is presented in S2 File.

The following analysis is based on a revision of the observational protocols that were available. It is our intention that it serves as a guide for researchers when considering specific risks of bias common to cohort and case-control designs in the measurement of the exposure of interest, measurement of the outcomes of interest, confounding and effect modification. The main biases are also noted in Table 1 for each of the standardized protocols and S1 Fig shows the overlap in risks of bias for the analytical observational study designs.

### Common biases in observational epidemiological studies

Although the randomized controlled trial (RCT) is the gold standard to measure causality in its unique advantage of random assignment, we must consider alternative research designs that permit relatively strong causal inferences. Therefore, when conducting observational studies researchers need to ensure that the internal validity of the study is not compromised by bias and that the results found are close to the “truth” [14, 27]. Biases common to all observational studies include selection bias and information bias (Table 2, Adapted from: Bonita et al. [28], Examples of low and high risk of bias in observational studies can be found in [26, 29]). Studies that attempt to show a causal association between an exposure and outcome can also be affected by confounding–these are the observational analytical designs, usually cohort and case-control studies.

**Table 2:** Common biases in observational epidemiological studies, along with the extent of their occurrence.

	Ecological	Cross-sectional	Case-control	Cohort
*Extent of*:				
Selection bias				
•Inclusion bias, self-selection	NA*	Probably	High	Low
•Loss to follow-up	NA	NA	Low	High
Information bias / measurement error				
•Recall bias	NA	High	High	Low
•Instruments	Probably	Probably	Probably	Probably
•Observer bias	Probably	Probably	Probably	Probably
Confounding	High	Probably	Probably	Probably

*NA–not applicable.

While all designs have their advantages and disadvantages, researchers need to be aware that depending on the research question [28, 30]:

- The probability of selection bias due to inclusion bias is highest in case-control studies.
- The probability of selection bias due to loss to follow-up is highest in cohort studies.
- The probability of measurement bias due to recall bias is highest in cross-sectional and case-control studies.
- Information bias due to instrument error and observer bias is common to all study designs.
- Confounding is an issue that is common to all observational analytical study designs.

### Measurement of the exposure of interest–challenges in accurately identifying ZIKV infection

The symptomatology of ZIKV infection includes fever, rash, and conjunctivitis, as well as other minor symptoms commonly associated with influenza, such as body aches and chills [31]. However, an individual may be infected with ZIKV but either have none or some of the aforementioned symptoms [1]. The range of symptoms of the ZIKV infection is so vast that a myriad of definitions have been generated as to what constitutes an infected individual (i.e. number and severity of symptoms present to qualify as a case of ZIKV), thus limiting the external validity of studies if they use variable criteria.

Perhaps the main critical issue for all observational studies conducted since the ZIKV outbreak of 2015 is the lack of a standardized definition of ZIKV infection due to challenges and limitations of available molecular and serologic diagnostic tests for ZIKV [32]. This can lead to risks of both selection bias and information bias due to misclassification of infection. In S1 Fig, this issue is depicted at the center of the diagram (E) at the intersection of the four analytical designs [17–20] described in Table 1, and also applies to the two descriptive observational protocols [21, 22]. In studies where ZIKV infection determines participation, there is a risk of selection bias if individuals do not meet the inclusion criterion for infection and may therefore be systematically excluded from participation. In studies where ZIKV infection does not determine participation, but is still considered an exposure of interest, there is a risk of information bias due to misclassification of exposure.

Serologic assays use certain biomarkers present in blood and other bodily fluids to test for the presence of antibodies suggestive of previous or ongoing ZIKV infection. However, immunology assays (immunofluorescence assay (IMF) and enzyme-linked immunosorbent assay (ELISA)) for ZIKV may result in misclassification errors due to cross-reactivity between ZIKV and dengue virus (DENV) or other flaviviruses co-circulating in the proximity of ZIKV patients and in addition the cross-reactivity but also the actual presence of anti-flavivirus antibodies for two different viruses in the same patient [5, 33]. At the time of writing, the United States Centers for Disease Control (CDC) stated that, “Due to serological cross-reactivity between flaviviruses, current IgM antibody assays cannot reliably distinguish between Zika and dengue virus infections” [5]. The lack of reliable distinction between ZIKV and DENV is a significant source of information bias for all studies of ZIKV where DENV is circulating, or has circulated, and could contribute to confounding of the association between exposure to the ZIKV and disease outcomes (see section 4 of this paper). However, a recent study using a novel anti-ZIKV ELISA based on recombinant ZIKV non-structural protein1 found that cross-reactivity with high-level dengue virus antibodies was not detected [34].

Interim case definitions for suspected, probable, and confirmed cases of zika have been defined by the PAHO/WHO and their use should enable standardization and help limit selection bias and information bias due to misclassification [35]. In the context of research confirmed cases are required. The standardized protocols reference the PAHO/WHO case definitions and include clear a description of which laboratory tests should be used during the course of infection and when, the procedures to be followed, and definitions of cases [36]. These include lab algorithms to rule out other flaviviruses, such as DENV and the collection of samples for all study participants, regardless of symptom status or case status. They also include recommendations for reporting of results stratified by symptom status. Further, all of the protocols include a standardized questionnaire [36] to ensure standardization of collection of information on symptoms and other relevant information. Questionnaires were specifically developed for each of the six zika standardized research protocols based on existing case report forms [16, 36].

While the test with highest specificity and sensitivity to detect ZIKV-infected patients is reverse-transcriptase polymerase chain reaction (RT-PCR), which detects the presence of ZIKV RNA in human body fluid samples [37, 38], the use of this method is limited as ZIKV can only be reliably detected in up to the first 7 days after infection [5, 32]. RT-PCR is expensive and samples must be sent to specialized laboratories for analysis, which are not yet readily accessible in all countries affected by ZIKV. Thus, for epidemiological studies, seropositivity is mostly defined as positive anti-ZIKV IgM antibodies AND plaque reduction neutralization (PRNT90) for ZIKV titers ≥20 and four or more times higher than for other flavivuruses; AND exclusion of other flavivirus [22]. Where the sensitivity and specificity of the serologic test is known, these can be adjusted for to correct for misclassification. Alternatively, sensitivity analysis can be undertaken using a range of plausible estimates for the misclassification. The testing strategy for patients presenting ≥ 7days after onset of symptoms focuses on IgM serology due to the availability of reagents. The WHO Interim Guidance on laboratory testing for Zika virus infection indicates that IgM detection should be performed for pregnant women in areas of endemic transmission or pregnant women who could have had contact with vector-borne or sexually transmitted Zika virus [39].

However, some issues remain and need to be addressed by the research community. These include development of better diagnostic tools; determination of the sensitivity, specificity and predictive value of serum ZIKV IgM; and assurance of reliable, accurate, and standardized testing [12, 40]. Other issues which are generally found with biomarkers are exacerbated by problems specific to the countries and regions where the zika epidemic is currently found. For example, there are limited labs with the capability of testing biological samples for ZIKV infection, which may lead to difficulties shipping samples to the labs in a timely manner and may put the integrity of the samples at risk due to the time of collection, maintaining of the cold chain, and material transfer issues [41]. Until more laboratories have the ability to test for ZIKV infection in biological samples, the possibility of reduced integrity of samples should be considered a potential source of information bias as it can lead to misclassification of exposure status [42].

### Measurement of the outcomes of interest—Challenges in defining the spectrum of diseases caused by ZIKV infection: Microcephaly and other congenital birth defects and Guillain-Barré Syndrome (GBS)

Measurement of the outcomes of interest is a risk of bias common to the observational analytical studies on ZIKV infection [17–20]. Research conducted to date has used a variety of case definitions for microcephaly, congenital ZIKV syndrome and GBS [12], which makes the comparison and pooling of results difficult.

*For microcephaly*–the International Fetal and Newborn Growth (INTERGROWTH) standard is considered the best method to classify a case of microcephaly in a fetus or newborn [43]. The WHO recognizes that there is a large variability in the levels of microcephaly present in different geographic populations, but it is likely that some of the variability is due to differences in measurement and subsequent classification, which would be a potential source of information bias [43]. Several methods can be used to determine head size, such as the ultrasound (during pregnancy), Magnetic Resonance Imaging (MRI) scan, and Computed Tomography (CT) scan, where available (see S1D Fig) [44].

*For Guillain Barré Syndrome*–WHO has published an interim guidance on the Identification and management of GBS syndrome in the context of ZIKV, which provides recommendations for clinical assessment based on the Brighton Collaboration criteria case definitions for GBS [45, 46]. In the standardized protocol relevant to GBS, the definition of a case is: GBS meeting levels 1–3 of diagnostic certainty for the Brighton Collaboration criteria case definitions for GBS [18, 45, 47].

Standardized and validated methods for measuring outcomes will help to prevent misclassification of outcomes leading to information bias. For the case-control study designs [17, 18], misclassification of outcome can also lead to selection bias, with those not meeting the inclusion criteria for a “case” and thus being systematically excluded from participation. To help overcome this issue clear definitions of cases are given in the ZIKV standardized protocols, though both protocols include a note that the studies will be used to refine and update recommendations for surveillance and case definitions [17, 18].

### Confounding and effect modification

In addition to selection and information bias due to challenges with currently available ZIKV diagnostic tests and clear definitions of key outcomes, for the analytical designs researchers need to account for potential confounders [17–20]. In order to prove causation between an exposure (in this case ZIKV infection) and an outcome (e.g., microcephaly in the newborn or GBS in adults) confounding needs to be minimized or, if possible, ruled out.

Confounding can occur when another exposure exists in the study population and is associated with both the disease and the exposure under study. Potential confounders include independent risk factors for the outcomes of interest, though to be confirmed as confounders they also need to be associated with the exposure of interest in the particular study and not be on the causal pathway between the exposure and outcome [28].

Potential confounders need to be considered at both the design and analysis stages of observational studies [28]. At the design stage, approaches that can be taken to prevent confounding include matching and restriction. Randomization is the best approach for preventing confounding but its use is limited to experimental designs. At the analysis stage confounding can be controlled by stratification or multivariate modeling. However, all of these approaches require that all potential confounders are identified and measured. Potential confounders are included as part of the standardized questionnaires for each of the standardized protocols and/or in the laboratory testing regime. In addition, for the case-control designs controls are matched to cases on some key variables.

In the case of microcephaly and other congenital birth defects of the newborn independent risk factors include certain infections during pregnancy, such as rubella, toxoplasmosis, syphilis, varicella-zoster, rubella, cytomegalovirus, and herpes infections (TORCH) in utero (see S1D Fig) [48]. Testing of serum for these potential confounders is recommended in the standardized protocols relevant for microcephaly. Other independent risk factors for microcephaly may include: severe malnutrition; maternal alcohol and tobacco use, maternal sociodemographic characteristics including age, race, education, marital status, body mass index and interruption of the blood supply to the baby’s brain during development and these are included in the standardized questionnaires [49, 50].

Given the possibility of misclassification of DENV and ZIKV, DENV should also be considered as a potential confounder in all cohort and case-control studies of ZIKV [12]. Questions to address exposures to these potential risk factors and others are included in the standardized questionnaires and/or in the laboratory testing regimes for the cohort and case-control studies for microcephaly [17, 19, 20].

For GBS, although the exact cause is unknown, most cases of GBS occur after a i) virus infection–such as the cytomegalovirus (a member of the herpes group), the Epstein-Barr virus or HIV; or ii) bacterial infection–such as infection from Campylobacter bacteria, a common cause of food poisoning [51]. Questions to address exposures to these potential risk factors and others are included in the standardized case-control study for GBS [18].

Despite the attempts to account for all potential confounders in the ZIKV standardized protocols, researchers using these protocols need to ensure that appropriate statistical analyses are undertaken to test and control for these. Some variables may also be modifiers of the effect and this need to be considered at the analysis stage of the study. Both users of the protocol and of the research should also be aware of unknown confounders.

### Risks of bias common to cohort designs of ZIKV infection

The risk of selection bias due to loss to follow-up is higher in cohort studies than in other observational designs (Table 2). This is particularly important if there is differential loss to follow-up due to the exposure. In the case of ZIKV infection, cohort studies are ideal for investigating multiple outcomes from a single exposure [52], which is applicable when the primary exposure of interest is ZIKV infection and there is a wide range of outcomes of interest [19, 20]. If performed in a conscientious and rigorous manner, cohort studies can be used to determine causality, given that exposure and outcome are investigated in a temporal framework [53], i.e. the exposure (ZIKV) clearly comes before the outcome (e.g. microcephaly or GBS).

The two main sources of selection bias in cohort studies arise during the recruitment and the follow-up phases (see S1G Fig). In the recruitment phase, the considerations regarding classification of ZIKV infection are similar to those for all observational designs as described in section 3. In addition, care is needed to ensure that the exposed and unexposed individuals are recruited from the same source population so that they are similar in all regards except for the particular exposure of interest [14, 52, 54]. If the exposed and unexposed individuals differ in extraneous factors (potential confounders), then internal validity is at risk and any differences observed in the outcome of interest may not be attributable to a causal relationship [14, 55], unless these factors can be controlled for in the analysis.

In the follow-up phase, time is crucial to minimize losses to follow-up. In the case of ZIKV infection, follow-up is important for analyzing causal considerations such as temporality, dose-response, and consistency [55]. The follow-up period must be long enough for a sufficient number of outcome events to occur or be observed, increasing the statistical power to detect differences between the exposed and unexposed individuals [56]. Depending on the type of outcomes sought, minimum length to follow-up may vary. However, even if intended follow-up times are appropriate for the outcomes of interest, loss to follow-up still represents a significant risk of selection bias and a threat to validity if there is differential loss between the exposed and unexposed cohorts. That is, if loss is classified as “missing at random”, then theoretically, there is no risk for selection bias, but if the loss is classified as “missing not at random”, then one must make the conservative assumption that the loss is related to exposure status, which then introduces selection bias [57].

#### Cohort study of ZIKV-infected patients to measure the persistence of ZIKV in body fluids

This is a prospective observational cohort study of men and women, aged 18 years and above, who have ZIKV positive RT-PCR blood or urine samples and their symptomatic or asymptomatic household contacts [21]. Participants will be followed for 12 months in order to evaluate the persistence of virus, reactivation and reinfection at regular intervals (9 visits in total following the baseline visit). The risk of selection bias and loss to follow-up in this study needs to be considered by researchers using the protocol and by users of the results. Given the frequency of visits and the type of measurements (blood and body fluids) there may be reluctance to participate and/or continue to final follow-up. This reluctance may be greater in asymptomatic household contacts than in the symptomatic index cases. In addition researchers need to be aware of the risk of bias in the measurement of the exposure (as detailed above).

#### Cohort study of pregnant women and newborns exposed to ZIKV during pregnancy

The main risks of bias in this study are in measurement of the exposure, outcomes and confounders (as detailed above). There is also a risk of selection bias due to possible underrepresentation of ZIKV negative and asymptomatic ZIKV positive women if they perceive that their babies are not at risk of fetal abnormalities. Bias due to loss to follow-up should also be considered.

For this study, pregnant women are recruited as soon as possible once the pregnancy has begun regardless of the development of symptoms, with follow-up visits at least once per trimester, ideally more often [20]. Measurements of the fetus or newborn are made at birth and newborn infants should be followed for a minimum of 1 month following birth [20] (see Cohort study of newborns for longer duration of follow-up). In this protocol IgM and IgG serological methods and real-time RT-PCR have been recommended.

Given the short duration of this study (approximately 10 months), the chance of loss to follow-up are relatively low. A concern however is that, given the severity of potential birth outcomes in babies exposed to ZIKV *in utero*, a mother may choose to terminate a pregnancy after learning of a probable or positive ZIKV exposure [58, 59]. To account for this, researchers using the ZIKV standardized protocol are requested to record the outcome of the pregnancy (whether miscarriage, termination or live birth), including any birth defects if detected. Among the Latin American countries (LAC) countries that are currently afflicted with ZIKV, there is a wide range of acceptability for the termination of a pregnancy, so the availability of that option would be highly dependent on the national and cultural context [60, 61]. The standardized ZIKV protocol provides guidance on information that can be provided to pregnant women if they are exposed, infected and/or if an abnormality is identified so that she and her partner can make informed decisions [20].

In the ZIKV standardized protocol, the frequency of follow-up is suggested to be at least once per trimester to allow for the analysis on the association between timing (trimester) of ZIKV infection in the mother and resulting frequency of abnormalities in the fetus. More frequent follow-up will allow researchers to obtain better data about the timing of appearance of congenital abnormalities in the fetus. Whatever the chosen frequency, this needs to be consistent between women both exposed and unexposed to ZIKV.

#### Cohort study of newborns and infants born to mothers exposed to ZIKV during pregnancy

In the event that a cohort study of pregnant women has also taken place in the region of study, it is strongly recommended and preferred (logistically and scientifically) to follow-up the newborn of mothers enrolled in that study [19] for a minimum of 2 years after birth. Ideally, the cohort study will follow the development of the children up to the age of 5 years, if resources permit [19]. The newborns of both ZIKV positive and negative women are recommended to be included for follow-up visits at 1, 3, 6, 9, 12, 18 and 24 months. At each visit, developmental evaluations will include assessments of epilepsy, hearing, vision, swallowing and spasticity/movement in the infant, following WHO guidelines.

Given the longer duration of this cohort study there is greater potential for loss to follow-up. If the loss to follow-up is greater for newborns without apparent abnormalities and/or related to ZIKV exposure this could introduce selection bias. There is also a risk of selection bias due to underrepresentation of babies born to asymptomatic women if infection is missed (see section 2). Researchers using the ZIKV standardized protocol need to be aware of this risk and make all reasonable efforts to maximize recruitment, follow-up and account for drop-outs. The risks of bias due to measurement of the exposure, outcomes and confounders (as detailed above) also apply to this protocol.

### Risks of bias specific to case-control designs

In addition to challenges in accurate diagnosis of ZIKV infection (section 2), the main source of bias common to all case-control studies of ZIKV infection occurs at the point of selection of cases and controls to be included in the study. Both cases and controls should represent the same base set or same source population [62]. That is, cases from one population should not be compared with controls arising from a different population. However, the cases and controls do not necessarily need to be representative of the total underlying source population, but to have a similar baseline level of risk [62].

The selection of controls is a particular challenge, and there are a few different ways to recruit them. Controls can be recruited from the general population, from amongst friends and family of the cases, or from the same hospital where cases were recruited [63]. However, care must be exercised when selecting controls from hospitals since they may have comorbid conditions or exposures that make them systematically different from the cases, increasing the risk of confounding [63–65].

The selection of controls provides an important opportunity to reduce the risk of confounding in the study design. Because the important outcomes of ZIKV infection are relatively rare, matching should be strongly considered. Matching is a method used to ensure comparability between cases and controls with regard to potential confounding factors. In an individual matching scheme, each case is matched to a control based on various potential confounding factors, such as age, sex, or other variables that are known to be associated with the outcome of interest [62, 65, 66]. However, over-matching can occur when cases and controls are matched by a non-confounding variable associated to the exposure but not to the disease, and may result in bias. Over-matching can underestimate associations and cannot be corrected in the analysis. Also, it is difficult to match for every possible confounder and unmatched confounders must still be considered in the analysis phases of the study.

Given the retrospective measurement of exposure status and potential confounders in case-control studies the risk of bias due to measurement error is also greater than in the prospective cohort studies. The advantage, however, of the case-control study is that smaller sample sizes are needed than for cohort studies and the follow-up time much shorter–which reduces the risk of selection bias due to loss to follow-up. The risks of bias due to measurement of the exposure, outcomes and confounders (as detailed above) also apply to these two protocols (17,18).

Effect modifiers (interaction variable) can over- or underestimate the risk of bias by modifying the observed effect of a risk factor on disease status. In the case of Zika, this includes the variable gestational age, and it is not recommendable to match for this type of variable as it is necessary to show the directionality and the magnitude of the modification of the effect granted.

## Conclusions

We have enumerated the potential risks of bias and confounding in all observational studies, which are applicable to observational studies of ZIKV. We have developed six standardized protocols for observational studies whose aim is to minimize bias by specifying clear criteria for selection of participants; standardized measurement methods and definitions for both exposures (ZIKV) and outcomes (microcephaly, GBS); and specification of potential confounders, along with standardized methods to measure and control for them. Concerted efforts must be made in the design and analysis stages of observational studies related to ZIKV infection. The use of these standardized protocols will help minimize potential risk of bias of future studies on ZIKV infection. The use of the standardized protocols by researchers in the field will increase the quality of their data. In addition, it will facilitate comparability of data and thus the possibility of performing joint analyses to answer complex questions that individual studies cannot answer yielding more reliable, valid and generalizable results. Users of the ZIKV research, including public health decision-makers, should critically appraise future observational studies for known risks of bias and use this information in their decision-making.

### Implications for policy and research

- Researchers undertaking ZIKV infection studies should use the standardized protocols and contribute to efforts to share and pool data.
- Funders of ZIKV research should prioritize research that uses these protocols and/or fills gaps in knowledge related to the protocol.
- Policy makers should use the information from research undertaken using the standardized protocols to guide decision making regarding public health advice and resource allocation.
- Researchers, policy makers and funders can help to improve the protocols by contributing to the generation of better information to calculate sample size, to determine the epidemiological spread of ZIKV and timing for research, expertise needed to run some tests, and legislation / regulations that enable the sharing of samples.

## Supporting information

S1 Fig Venn-diagram showing the risks of bias as they relate to the analytical observational study designs. Circles represent the different study designs. A: represents the risk of bias common in case-control studies for microcephaly and for the cohort of newborns; B: represents the risk of bias common in both case-control studies and for the cohort of newborns; C: represents the risk of bias common in both case-control studies; D: represents the risk of bias common in the case-control study for microcephaly and both cohort study designs; E: represents the risk of bias common in all four study designs; F: represents the risk of bias common in both case-control studies and the cohort of pregnant women; G: represents the risk of bias common in both cohort studies; H: represents the risk of bias common in both cohort studies and the case-control study of Guillain-Barré Syndrome; I: represents the risk of bias common in the case-control study of Guillain-Barré Syndrome and the cohort of pregnant women. Risks of bias were found for D, E and G. No specific risks of bias were detected for A, B, C, F, H, I.
(TIF)

S2 Fig Flow diagram of the search and protocol selection. (TIFF)

S1 File Protocols identified through a systematic search of clinical trial databases. (PDF)

S2 File Prisma Checklist. (DOC)

## Abbreviations

CDC: Centers for Disease Control
CONSISE: Consortium for the Standardization of Influenza Seroepidemiology
CT: Computed Tomography
ELISA: Enzyme-linked immunosorbent assay
GBS: Guillain-Barré Syndrome
IMF: immunofluorescence assay
INTERGROWTH: International Fetal and Newborn Growth
ISARIC: International Severe Acute Respiratory and Emerging Infection Consortium
LAC: Latin American countries
MRI: Magnetic Resonance Imaging
PAHO: Pan American Health Organization
PCR: polymerase chain reaction
TORCH: includes Toxoplasmosis, syphilis, varicella-zoster, parvovirus B19, Rubella, Cytomegalovirus (CMV), and Herpes infections
WHO: World Health Organization
ZIKV: Zika virus
