# Correction: Risk of bias and confounding of observational studies of Zika virus infection: A scoping review of research protocols

**DOI:** 10.1371/journal.pone.0189027

**Published:** 2017-11-30

**Authors:** Ludovic Reveiz, Michelle M. Haby, Ruth Martínez-Vega, Carlos E. Pinzón-Flores, Vanessa Elias, Emma Smith, Mariona Pinart, Nathalie Broutet, Francisco Becerra-Posada, Sylvain Aldighieri, Maria D. Van Kerkhove

The Copyright statement appears incorrectly. The correct statement is: © 2017 Pan American Health Organization; licensee “PLOS ONE”. This is an open access article distributed under the terms of the Creative Commons Attribution IGO License (https://creativecommons.org/licenses/by/3.0/igo/legalcode), which permits unrestricted use, distribution, and reproduction in any medium, provided the original work is properly cited. In any reproduction of this article there should not be any suggestion that PAHO or this article endorse any specific organization or products. The use of the PAHO logo is not permitted. This notice should be preserved along with the article’s original URL.

The following information is missing from the Competing Interests section: The views expressed in this article are those of the authors and do not necessarily represent the decisions, policies, or views of the WHO or PAHO.
