# Supplementary figures and images for: Risk of bias and confounding of observational studies of Zika virus infection: A scoping review of research protocols
Source: PLoS One. 2017 Jul 7;12(7):e0180220. doi: 10.1371/journal.pone.0180220 (PMC5501456; doi:10.1371/journal.pone.0180220)

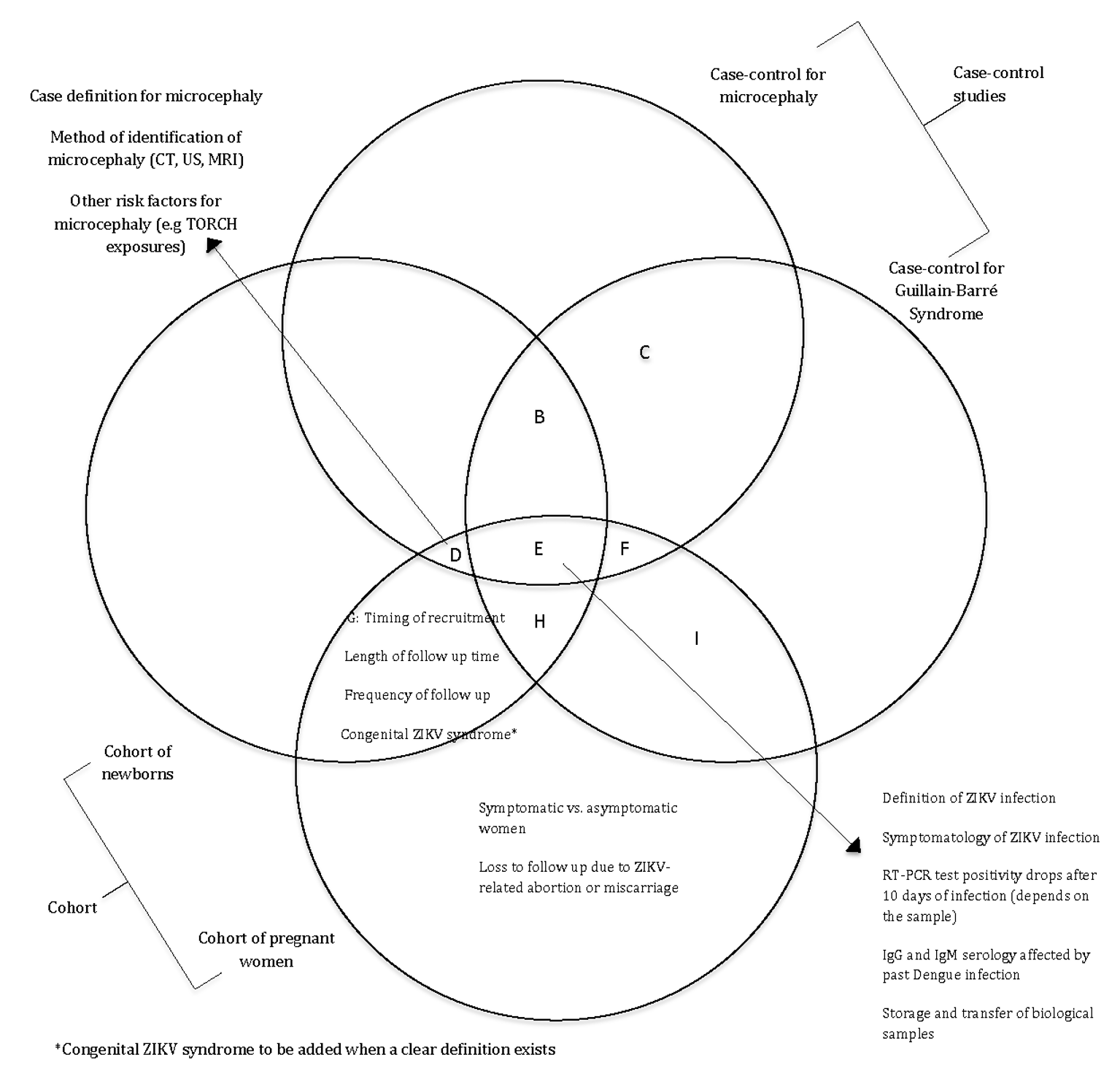

Supplement: S1 Fig — Circles represent the different study designs. A: represents the risk of bias common in case-control studies for microcephaly and for the cohort of newborns; B: represents the risk of bias common in both case-control studies and for the cohort of newborns; C: represents the risk of bias common in both case-control studies; D: represents the risk of bias common in the case-control study for microcephaly and both cohort study designs; E: represents the risk of bias common in all four study designs; F: represents the risk of bias common in both case-control studies and the cohort of pregnant women; G: represents the risk of bias common in both cohort studies; H: represents the risk of bias common in both cohort studies and the case-control study of Guillain-Barré Syndrome; I: represents the risk of bias common in the case-control study of Guillain-Barré Syndrome and the cohort of pregnant women. Risks of bias were found for D, E and G. No specific risks of bias were detected for A, B, C, F, H, I. (TIF) [file pone.0180220.s001.tif]

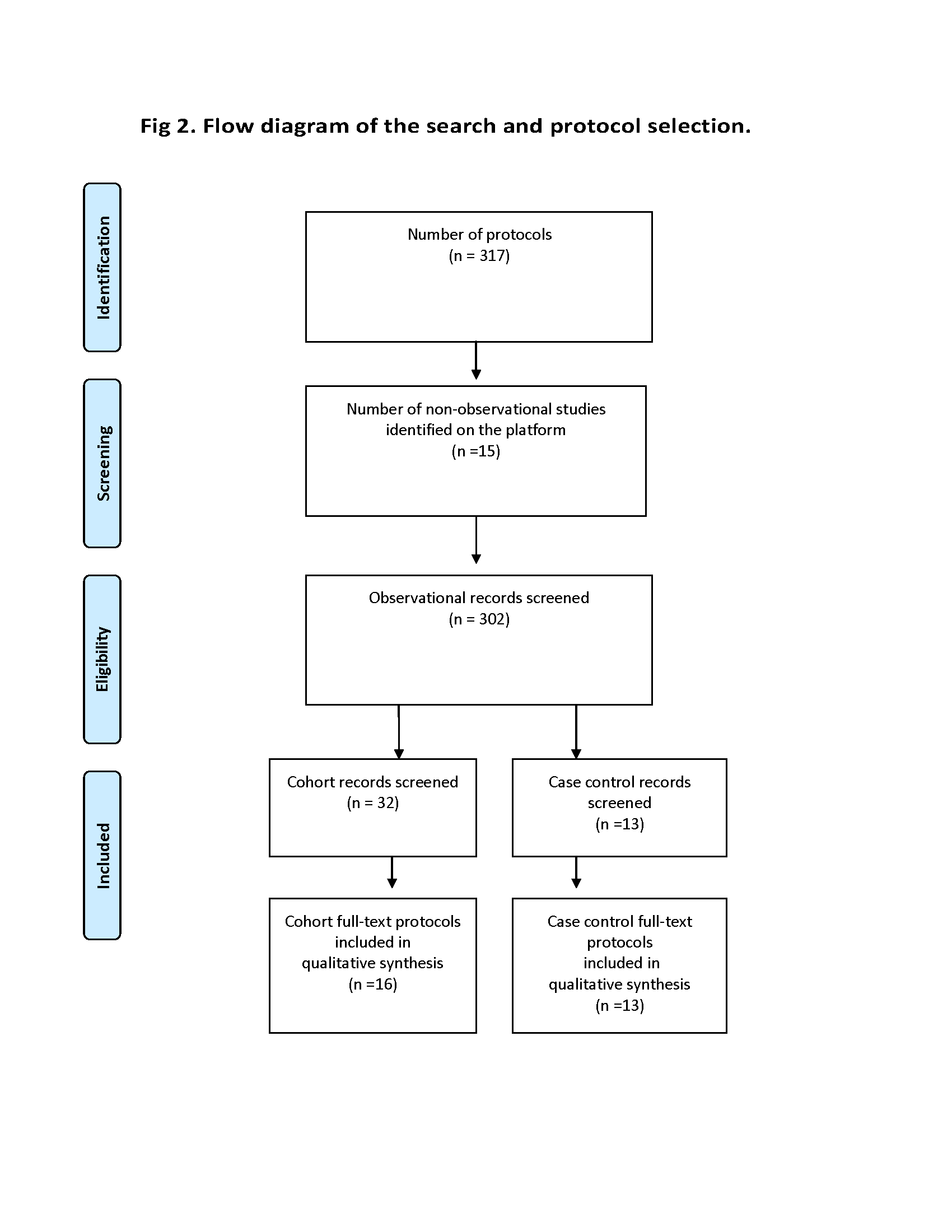

Supplement: S2 Fig — (TIFF) [file pone.0180220.s002.tiff]
