# Supplementary material for: Risk of bias and confounding of observational studies of Zika virus infection: A scoping review of research protocols
Source: PLoS One. 2017 Jul 7;12(7):e0180220. doi: 10.1371/journal.pone.0180220 (PMC5501456; doi:10.1371/journal.pone.0180220)
Supplement: S1 File — (PDF) [file pone.0180220.s003.pdf]

S1 File: Protocols identified through a systematic search of clinical trial databases

International Clinical Trials Registry Platform (ICTRP)

Sistema Nacional de Ética em Pesquisa (SISNEP)

Sistema de Informação da Rede Brasileira de Avaliação de Tecnologias em Saúde (SISREBRATS)

Pesquisa Saúde (Pesquisa SUS), Conselho Nacional de Desenvolvimento Científico e Tecnológico (CNPq)

Comissão da Coordenação de Aperfeiçoamento de Pessoal de Nível Superior CAPES, Ministério da Ciência, Tecnologia e Inovação

Registro peruano de ensayos clínicos y de estudios observacionales

Registro nacional de investigaciones en salud (RENIS)

Registro nacional de ensayos clínicos (RNECCOFEPRIS)
